# Cyanobacterial Biofertilizer Production by Guanidine-Producing Enzymes

**DOI:** 10.1021/acssynbio.5c00801

**Published:** 2026-02-10

**Authors:** Hakyung Lee, Jacob Sebesta, Eric Schaedig, Chao Wu, Himadri B. Pakrasi, Jianping Yu

**Affiliations:** † Bioscience Center, 53405 National Laboratory of the Rockies, Golden, Colorado 80401, United States; ‡ Washington University, Saint Louis, Missouri 63130, United States

## Abstract

Cyanobacterial production of a biofertilizer shows promise as an environmentally benign alternative to conventional nitrogen fertilizers, reducing environmental and energy burdens through light-driven nitrogen and carbon fixation. One route to realizing the potential for a nitrogen-rich, slow-releasing biofertilizer involves the genetic engineering of cyanobacteria to produce guanidine. Recent advances have demonstrated enzymatic guanidine production in cyanobacteria, but an understanding of cyanobacterial guanidine metabolism is still limited. This Perspective highlights strategies and opportunities for cyanobacterial guanidine production in a Design–Build–Test–Learn cycle. Exploring new guanidine-producing enzymes via phylogenetics could expand candidate enzymes, while understanding the metabolism of substrates can identify constraints and opportunities in substrate utilization. Additionally, guanidine sensing and export are crucial areas of study to enable continuous fertilizer production and stable nitrogen flux. These strategies will guide the development of advanced nitrogen biofertilizer strategies for the agricultural sector.

## Graphical abstract

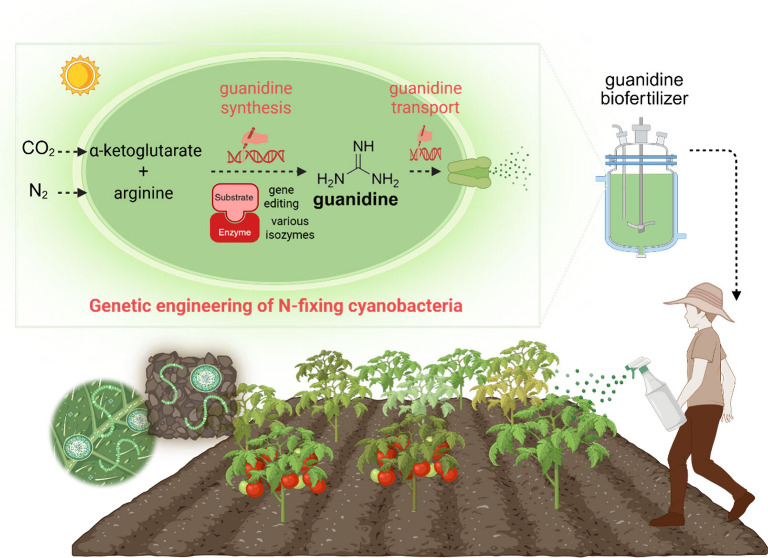

## Introduction

To enhance agricultural productivity and address the global food demand, fertilizers play a crucial role by providing vital nutrients for plant growth.^1,2^ Among these nutrients, nitrogen is the most critical element.^3^ Currently, synthetic nitrogen fertilizers are produced through the energy-intensive Haber-Bosch process, highlighting the urgent need for sustainable fertilizer alternatives.^4,5^ Cyanobacterial biofertilizers have gained recognition as promising alternatives to traditional chemical fertilizers owing to their ability to convert atmospheric gases into valuable organic compounds.^6–8^ Certain cyanobacteria fix nitrogen via the enzyme nitrogenase, which facilitates the transformation of atmospheric nitrogen gas into ammonium. These nitrogen-fixing cyanobacteria also contribute to soil agglomeration and fertility by enhancing soil porosity, thereby improving water–air circulation, conserving moisture within their cells, and releasing beneficial substances into the soil.^7,9^ Recognizing the potential of biofertilizers, relevant industries are actively advancing microbial biofertilizer technologies. Current research includes genetic engineering aimed at improving biological nitrogen fixation and fertilizer efficiency, exemplified by companies such as Pivot Bio, Kula Bio, and Azotic Technologies,^10–13^ as well as applications involving nitrogen-fixing consortia developed by Algaenite and BioConsortia.^14^

Existing nitrogen-containing fertilizer products on the market today include ammonium, urea, and several other compounds; however, these compounds possess drawbacks (Table 1). To address these issues, we have investigated alternative nitrogen fertilizers that can be produced via cyanobacteria. Ideally, a biologically produced nitrogen fertilizer should be an organic, nitrogen-rich, and enzymatically labile molecule. Guanidine, which contains 71.1% atomic nitrogen by mass, exhibits superior fertilizer efficiency compared to other nitrogen compounds due to its slow-release properties.^15,16^ Plants absorb guanidine through their roots from the soil,^17^ and it shows a higher uptake rate than other nitrogen sources.^18^ The specific mechanism of guanidine utilization in plants has not yet been studied, presenting an interesting opportunity for flux analysis to explore the path of isotope-labeled guanidine within plant cells. Unlike conventional nitrogen fertilizers such as urea and nitrate, which are typically converted to ammonium during nitrogen metabolism, guanidine remains in its original form within plants as a downstream molecule in inorganic nitrogen metabolism.^17^ Its gradual degradation into urea and ammonia by guanidine-degrading enzymes ensures a supply of a simpler form of nitrogen during crop growth. Additionally, guanidine exhibits a lower volatility than ammonia, which may enhance nitrogen retention in farmlands and reduce associated costs. A study involving mutant plants capable of possessing guanidine, including *Robinia* species, *Alfalfa*, *Broccoli*, and *Arabidopsis*, showed seedling viability in guanidine presence, indicating that these plants can tolerate guanidine.^19^

**Table 1:** Summary of Nitrogen Fertilizers

Type	N (wt %)	Benefits and considerations	Application
Ammonium nitrate	35	Continuous nitrogen supply through direct nitrate and indirect ammonium applications.	Spraying granules, watering near the root zone of the plant.
		Potential explosion caused by nitrogen oxide gas and water.^20^	
Ammonia (anhydrous)	82	Cost-efficiency due to its high nitrogen content.^21^	Injecting gas into soil.^21^
		Potential nitrogen loss due to high volatility and temperature sensitivity.	
Ammonium sulfate	21	24% sulfur further aids plant growth.	Direct spreading to soil or a foliar spray.
		Ammonia loss depending on the pH.^22^	
Calcium ammonium nitrate (CAN)	27	Continuous nitrogen supply from direct nitrate and indirect ammonium.	Direct spreading to soil or dripping irrigation.
		Potential emissions.^23^	
Calcium cyanamide	19.8	Slow-release fertilizer.	Spreading granular fertilizer onto soil.
		European Union has proposed its restrictions due to health concerns.	
Urea	46	Cost-efficiency due to its high nitrogen content.	Foliar spray because of high solubility and leaf absorption.
		Potential ammonia loss to the air.	The slow-release method is being developed.^24,25^
Struvite	5.7	Slow-releasing fertilizer with low water solubility.	Mixing into soil.
		Mainly used as P source and low N content.	
Arginine	32.1	More efficient uptake than inorganic forms.^26^	Not applicable due to high cost.
		Lower nitrogen content.	
Guanidine	71.1	High nitrogen content, slow-release properties, and lower volatility than ammonia.	Living cyanobacteria directly – This study

To date, microbial production of guanidine remains underexplored. Cyanobacteria do not naturally produce guanidine. Proposed integration of known guanidine-forming enzymes into a cyanobacterial genome results in simplified nitrogen metabolism pathways, as shown in Figure 1. Fixed nitrogen is converted to ammonium by nitrogenase, and photosynthetic carbon fixation generates alpha-ketoglutarate (AKG). AKG is used for nitrogen assimilation via the glutamine synthase-glutamate synthase (GOGAT) cycle. Both glutamate and AKG interface with a proposed guanidine biosynthesis (GUB) cycle^27^ where they are used to generate arginine. Guanidine-forming enzymes catalyze the degradation of arginine into guanidine and P5C, and P5C is recycled to glutamate via P5C dehydrogenase (Figure 1).

**Figure 1:**
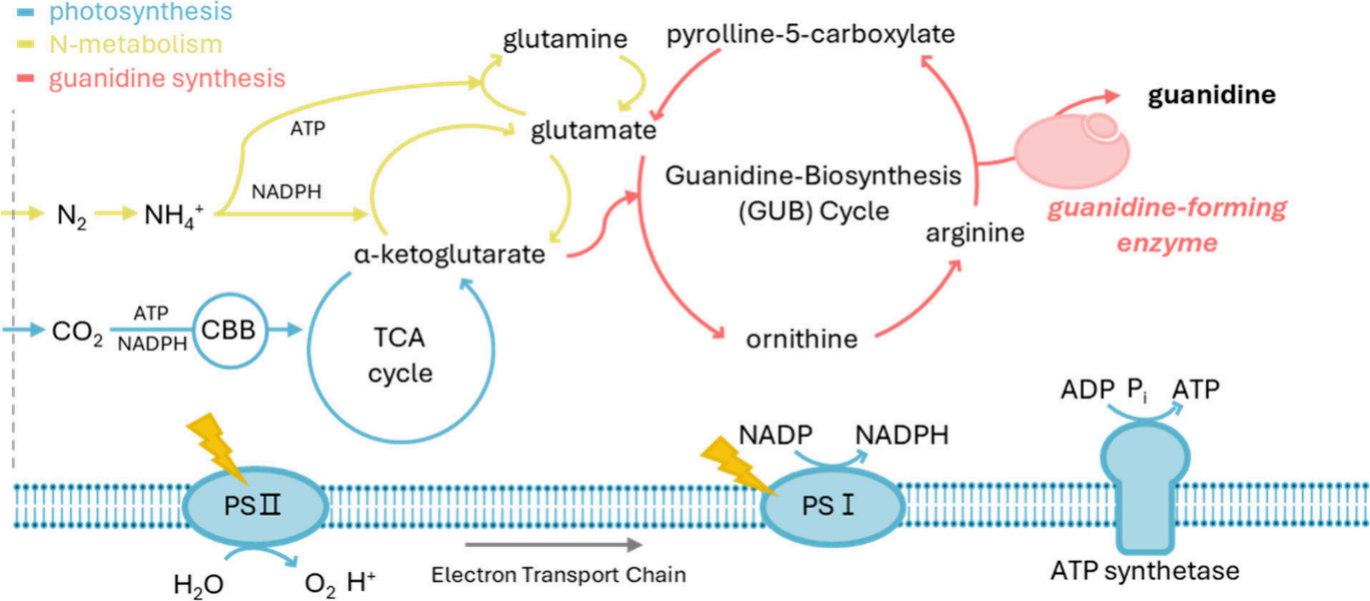
Scheme of enzymatic guanidine synthesis in cyanobacteria system including atmospheric nitrogen, carbon fixation, and photosynthesis.

Researchers have introduced guanidine-producing enzymes into cyanobacteria in a few examples using model species such as *Synechocystis* sp. PCC 6803 and *Anabaena* sp. PCC 7120.^27,28^ This area remains largely uncharted, underscoring the need for innovative approaches to achieve efficient guanidine biosynthesis in cyanobacteria and address sustainability challenges in agriculture. This process requires coordinated advances across the Design–Build–Test–Learn (DBTL) cycle. A critical aspect of biosynthesis involves regulating guanidine levels as excessive intracellular concentrations are toxic. Guanidine exhibits toxicity,^29^ impairing various cellular metabolic functions and damaging essential proteins. Additionally, guanidinium interferes with enzymatic activities, including arginase, due to competitive interactions stemming from structural similarities between arginine and guanidine.^29,30^ This interference disrupts nitrogen storage and utilization machinery. Consequently, controlling guanidine concentrations within cyanobacterial cells is vital to maximize the product yield and minimize toxicity. In this perspective, we outline potential genetic engineering strategies to enhance cyanobacterial guanidine production, within a DBTL framework, highlighting opportunities for innovation and future research directions

## Conceptual Strategies for Engineering Cyanobacteria to Produce Guanidine

The first steps in designing a cyanobacterial guanidine-producing system involve discovering suitable enzymes and identifying the key catalytic steps and potential bottlenecks, which will guide rational design choices. To date, multiple enzymes capable of producing guanidine have been identified. The initial demonstration of Ethylene-Forming Enzyme (EFE), which can synthesize ethylene, involved recombinant expression of the gene from *Pseudomonas syringae* pathovar PK2.^31^ The reaction catalyzed by this Fe­(II)/2-oxoglutarate dependent oxygenase (2-ODD) proceeds as follows: AKG + 3 O_2_ + l-arginine → 2 ethylene + succinate +7 CO_2_ + guanidine + P5C.^32^ During this process, guanidine appears as a reaction product of arginine oxidation in a 1:2 ratio when EFE was expressed in *E. coli*. Consistent with this observation, Wang et al. reported guanidine formation in cyanobacteria via the EFE reaction, coining the term, Guanidine Biosynthesis (GUB) Cycle to describe the collection of reactions apparently operating to generate guanidine from ammonium and CO_2_ and recycle P5C.^27^ This GUB cycle does not impose a significant metabolic burden on cyanobacteria. In a strain where EFE redirects about 10% of the fixed carbon to ethylene production, the growth rate was nearly identical with that of the wild type. The overall levels of ATP and NADPH generation and consumption increased by nearly 20%. Carbon flux toward AKG increased 3-fold to support ethylene production.^33^ Although direct experimental evidence is lacking, it is plausible that metabolic plasticity may also apply to nitrogen flux to enable a higher rate of arginine/guanidine production, which deserves further investigation.

Although EFE has demonstrated the capacity to synthesize guanidine within cyanobacterial systems, variants of EFE or related enzymes within the same family may have superior enzymatic properties. A few prior studies have identified enzymes with lower ethylene production compared to P5C production, which may imply that higher guanidine synthesis efficiency has been completed. Critical residues and coordination environments that govern the activity of enzymes within the 2-ODD family provide valuable insights into the mechanisms underlying guanidine production. This enzyme family uses iron­(II) as a cofactor. Arginine and AKG are the main substrates. They bind near the iron center within the active site of the 2-ODD family of enzymes. Binding causes significant conformational changes in the enzyme’s active site.^34^ Martinez et al. examined several mutants of the *P. syringae* EFE cocomplexed with different metals and substrates to elucidate the reaction mechanism.^35^ The observed changes in reaction partitioning between ethylene and P5C formation among these variants suggest the existence of differing guanidine production levels. Furthermore, other enzymes capable of producing guanidine have been identified. For instance, Din11, a member of the 2-oxoglutarate-dependent dioxygenase (2-ODD) subfamily C in *Arabidopsis*, recently demonstrated guanidine production from arginine.^19^ Additionally, the enzyme known as NapI, an arginine-4,5-desaturase, catalyzes the desaturation of the C_4_–C_5_ bond of arginine during the biosynthesis of naphthyridinomycin, yielding guanidine via an unstable precursor, 5-hydroxyarginine.^36^ These findings suggest that studying novel enzymes within the 2-ODD family could reveal novel capabilities for guanidine biosynthesis among these understudied enzymes. Broadening research efforts to include bacterial and eukaryotic organisms may uncover additional species capable of reactions analogous to those catalyzed by EFE and other characterized enzymes within the 2-ODD family, which may have improved kinetics or higher guanidine to ethylene ratios (Figure S1).

Efficient enzymatic guanidine production depends on the sufficient substrate availability. Strategies to stimulate substrate generation, such as pathway optimization and flux enhancement, are crucial during the design phase of an engineered system. The availability of these substrates, which can become rate-limiting, significantly influences the overall yield. Cyanobacteria possess the capacity to synthesize these substrates *in vivo* (Figure 2). Arginine is synthesized through nitrogen compounds generated via N_2_ fixation or uptake, followed by the ornithine-arginine-citrulline (OAC) cycle, whereas AKG is an intermediate in the TCA cycle. Engineered cyanobacteria have shown metabolic plasticity with increased carbon flow into the TCA cycle, replenishing the AKG pool. Increased AKG availability promotes the glutamine synthetase/glutamate synthase (GS-GOGAT) cycle, which could lead to more arginine synthesis.

**Figure 2:**
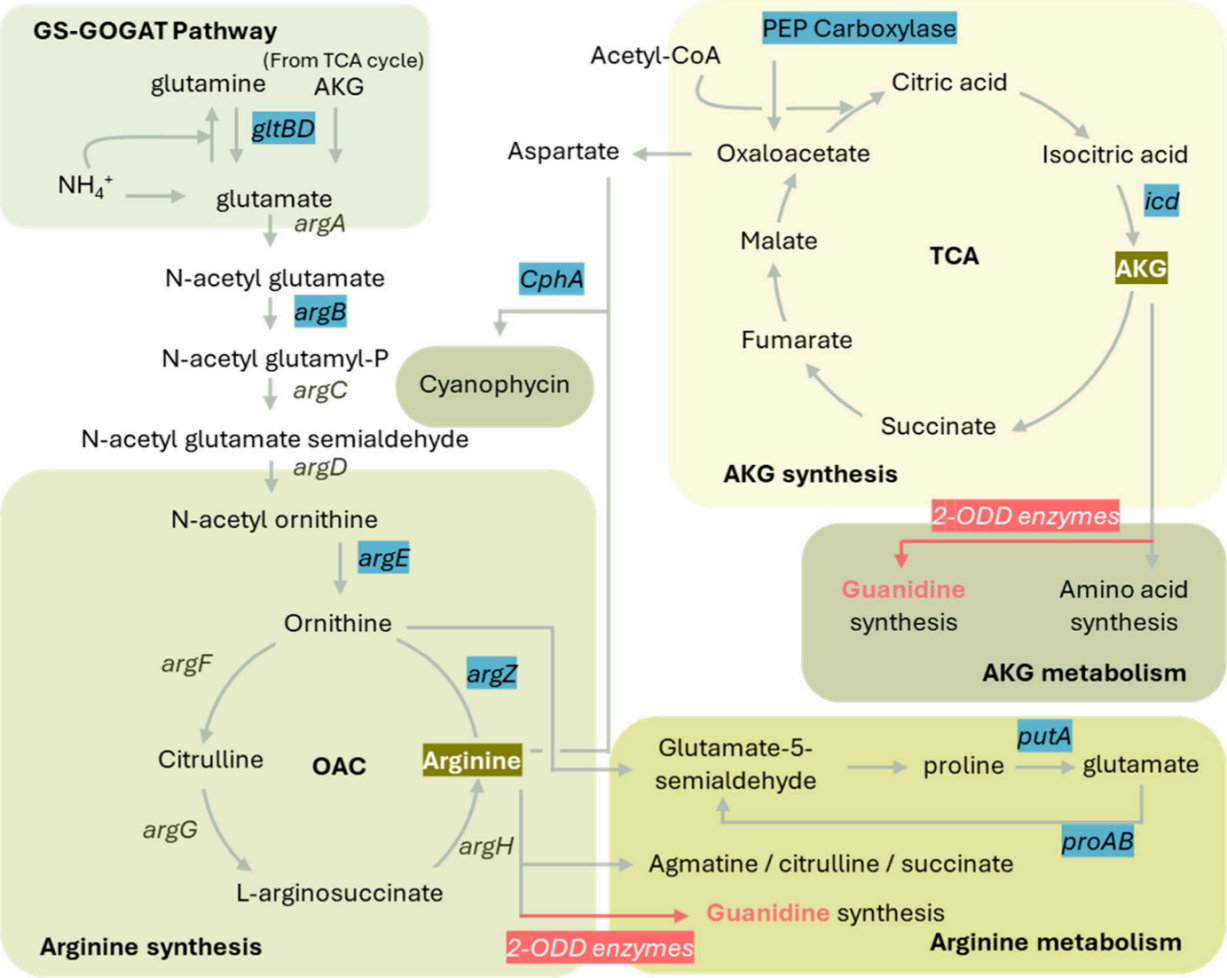
Critical enzymes involved in the synthesis of arginine and alpha-ketoglutarate (AKG), guanidine, as well as their utilization as potential candidates for genetic engineering. Potential target genes/enzymes are highlighted in blue, while guanidine-synthesizing genes are highlighted in pink. Two 2-ODD enzymes are identical but are separated to show their involvement in arginine and AKG metabolism.

To augment guanidine production, it is essential to exploit the metabolic adaptability of cyanobacteria by modulating metabolic fluxes to favor guanidine synthesis. First, to increase AKG availability, overexpression of isocitrate dehydrogenase (IDH) has the potential to increase AKG availability within the TCA cycle by converting additional isocitrate to AKG. Moreover, phosphoenolpyruvate carboxylase (PEPC) catalyzes the carboxylation of PEP into oxaloacetate, offering an energy-efficient alternative pathway that bypasses primary oxidative routes.^37^ Regulation of these pathways must also be considered. The global signaling protein P_II_ plays a pivotal role by modulating the activities of *ntcA* and PEPC according to intracellular levels of AKG and the ATP/ADP ratio. When the AKG to nitrogen ratio declines under conditions of sufficient energy, P_II_ interacts with PEPC to promote carbon flux toward AKG.^38^ Overexpression of PEPC leads to increasing the ethylene production in the engineered *Synechocystis* strain expressing EFE, suggesting a corresponding potential rise in guanidine production.^37^ Regarding the other substrate, arginine, it is synthesized through a two-stage process: the first converts glutamate into ornithine, and the second transforms ornithine into arginine via the ornithine-ammonia cycle (OAC).^39,40^ Once glutamate is synthesized from fixed nitrogen through the GS-GOGAT pathway, it is fed into the arginine biosynthesis pathway. Glutamate is then transformed into ornithine through several enzymatic reactions. Flux through those reactions is regulated by the binding of P_II_ protein to N-acetyl-l-glutamate kinase (NAGK), or ArgB, which enhances arginine synthesis when nitrogen is ample.^41^ Subsequently, the OAC process begins with ornithine as the starting material. The key enzymatic steps include l-arginine decarboxylase (ArgZ), arginosuccinate lyase (ArgH), arginosuccinate synthase (ArgG), and ornithine carbamoyltransferase (ArgF). Regarding arginine utilization, ArgZ plays an important role in regulating intracellular arginine levels by converting synthesized arginine back to ornithine through its dihydrolase activity. Deletion of argZ may be one strategy to increase the intracellular arginine concentration available for guanidine synthesis. However, if not fully consumed by guanidine production, the effect of higher arginine concentrations on other regulated pathways, including PEPC regulation by P_II_ could reduce nitrogen assimilation. Several other genetic interventions may further alter arginine and glutamate metabolism to support guanidine production. Deletion or downregulation of glutamate synthase (*gltBD)* remove a competing pathway for AKG, thereby potentially enhancing guanidine production.^42^ Additionally, knockout of *putA* prevents the synthesis of glutamate from arginine, ornithine, or proline, allowing these precursors to be diverted toward guanidine biosynthesis.^43^ The gene *proB* encodes glutamate-5-kinase, which converts glutamate into glutamate-5-phosphate (G5P). Then, *proA* encodes glutamate-5-semialdehyde dehydrogenase (GDH), which reduces G5P to glutamate-5-semialdehyde. Deleting *proAB* is expected to boost guanidine production by limiting proline synthesis and shifting the flux to guanidine-producing enzyme.^44^ Besides biosynthesis and degradation, cyanobacteria store excess nitrogen as cyanophycin, a biopolymer that releases free arginine and aspartate upon depolymerization.^45,46^ Regulation of cyanophycin synthetase (*CphA*) can modulate its synthesis, thereby diverting more arginine from guanidine production.

## Genetic Tools for Modulating Guanidine Production and Export

Implementing the designed pathways requires a strong toolkit of genetic and cellular engineering strategies. When developing these tools, it is important to carefully evaluate biomass growth and photosynthesis. To attain high product yields with a low metabolic strain, various strategies can be employed. High-level approaches, such as transporter engineering, promoter regulation, and host organism selection, provide the foundation for building systems capable of controlled guanidine production and export. Developing a guanidine-based biofertilizer involves more than merely synthesizing guanidine within cyanobacteria. Another crucial component includes enhancing its extracellular guanidine availability for practical application. We discussed above the investigation of various enzymes and gene engineering targets to facilitate effective substrate supply. While a higher yield of guanidine is desirable, its buildup in cells can cause toxicity in cyanobacteria, harming pigment metabolism and leading to protein denaturation.^47^ Thus, it is essential to understand the guanidine metabolism and transport within these organisms (Figure 3).

**Figure 3:**
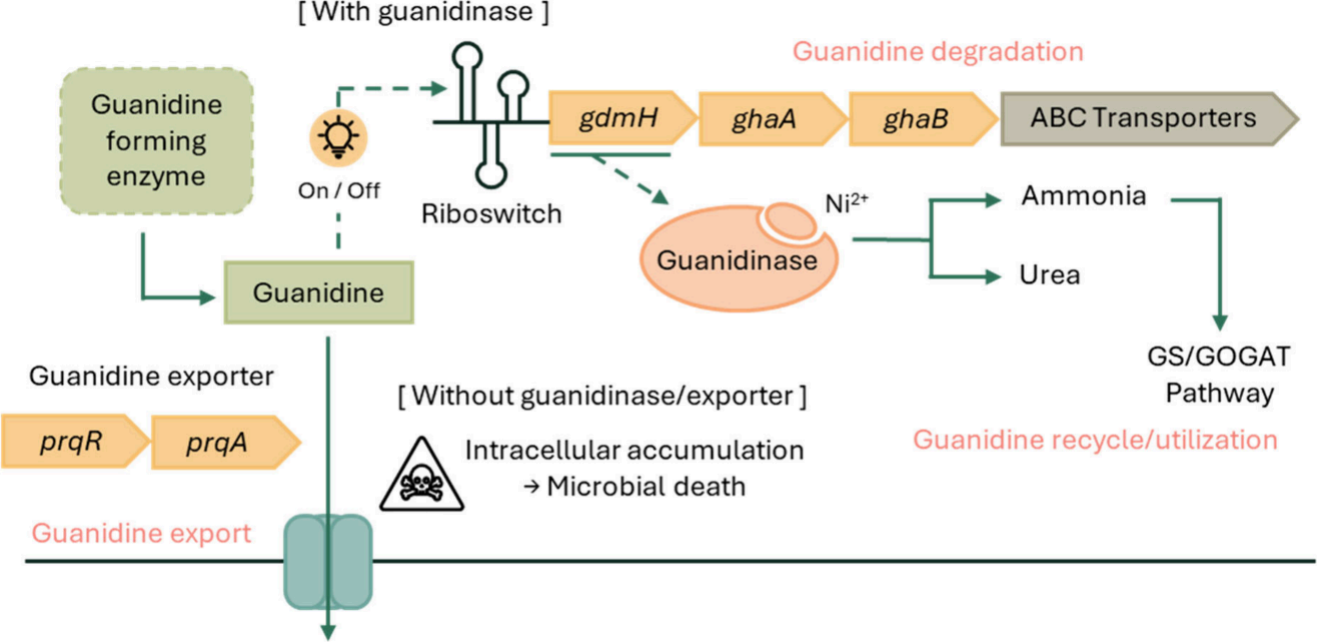
Metabolism of guanidine within cells produced by guanidine-forming enzymes.

Several cyanobacteria possess natural pathways that convert guanidine to ammonia via either urea or carboxyguanidine and allophanate. Once guanidine is synthesized, guanidine-dependent riboswitches can turn on and off guanidine degrading genes that help maintain cellular guanidine homeostasis.^48–51^ In *Synechocystis* sp. PCC 6803, a Ni^2+^-dependent guanidine hydrolase (GdmH) hydrolyzes guanidine into urea and ammonia, with its maturation proteins (GhaA and GhaB) are encoded in the operon.^51^ Additionally, an ABC transporter for importing guanidine is found within the same operon, enabling the use of guanidine for microbial growth. To maximize guanidine production, efficient export of guanidine rather than degradation is needed. Some cyanobacteria possess native exporters, while for others, heterologous expression of transporters can be employed. Recently, a native cyanobacterial multidrug efflux transporter, PrqA, was shown to be capable of guanidine export.^29^ The transporter of *E. coli* called Gdx, encoded by *sugE*, can also facilitate guanidinium ion (Gdm+) export while excluding other polar guanidinylated due to the hydrophobic nature of the transmembrane^30,53^ (Figure 4A). Since the efflux pump impacts the membrane system, it is critical to achieve successful guanidine export while minimizing cellular damage for cell stability.

**Figure 4:**
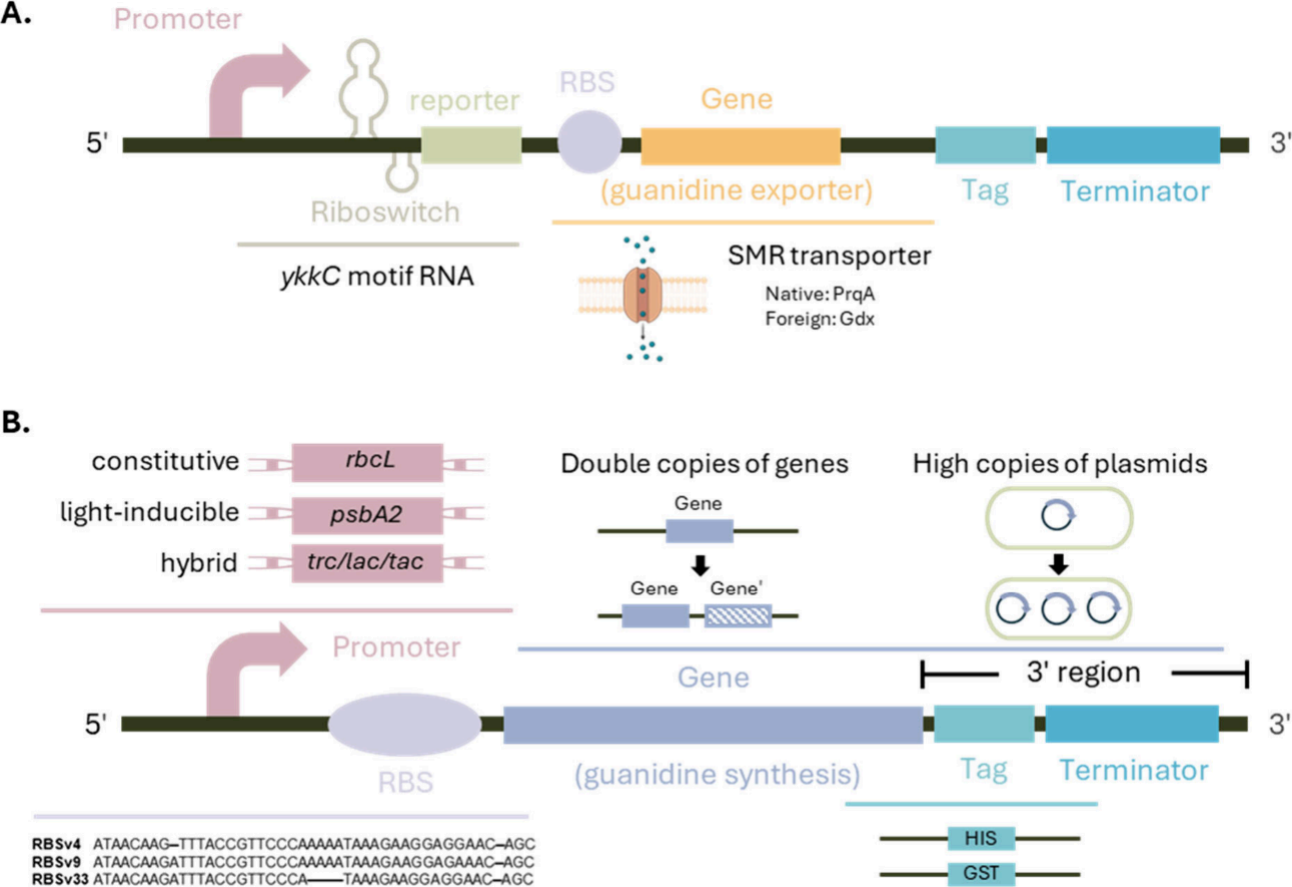
Synthetic biological guanidine regulation approaches: (A) Riboswitch-mediated guanidine transport system to alleviate the intracellular toxicity; (B) Regulatory elements involved in controlling the expression level of guanidine synthesis gene.

Furthermore, enzymatic guanidine production can be optimized, and gene expression levels can be balanced to preserve cellular health by modifying components within the expression system, such as the promoter, ribosome binding site (RBS), the gene itself, and terminators.^54,55^ Initially, adjustments may focus on modulating the expression of genes responsible for guanidine synthesis (Figure 4B). A straightforward approach involves increasing gene dosage by employing high-copy number plasmids with efficient replication origins or integrating multiple gene copies into the chromosomal DNA of hosts.^56,57^ Exploring different promoters or incorporating additional copies of the same promoter may achieve the desired level of gene expression. Candidates for constitutive promoters include the *rbcL* regulating RuBisCo expression or native *Synechocystis* promoters such as light-inducible *psbA2* and nitrogen-inducible *nit1*, which are suitable depending on the context.^58–69,60^ Inducible promoters enable a two-phase production strategy by toggling the guanidine synthesis pathway on and off. During the growth phase, guanidine production is off; once cells reach a robust state and no longer compete for maximum photosynthesis, the pathway is activated. For conditions requiring reduced expression, native promoters are preferable. Additionally, hybrid promoters such as *trc*, *lac*, and *tac* from *E. coli* are commonly utilized to attain a robust expression system.^61,62^ Depending on the objective, the 5′-untranslated region (UTR) length can be adjusted: shorter for efficient translation initiation or longer to accommodate added regulatory elements. From the translation perspective, an optimal 5′-UTR and RBS influence the targeted protein synthesis level.^63^ Design software tools are available to predict RBS sequences; however, these tools have demonstrated limitations in accuracy for cyanobacteria owing to organism-specific characteristics.^64,65^ Despite this limitation, prediction tools can aid in creating an RBS library for manual testing of various RBSs to evaluate guanidine production. Furthermore, tagging genes with a His-tag or GST-tag can streamline protein purification. Lastly, terminators ensure complete termination of the transcription.

Given these strategies, selecting the appropriate chassis for genetic engineering is a crucial step based on various factors. Important considerations include genetic tractability such as available annotated genomes, gene editing tools, transformation methods, and promoters, along with physiological traits like growth rate and robustness, and biosafety concerns like nonpathogenicity. Consequently, model cyanobacteria are promising starting points for guanidine bioproduction. Non-nitrogen-fixing options include *Synechocystis* sp. PCC 6803 and *Synechococcus elongatus* PCC 7942. For nitrogen-fixing bacteria, candidates, such as *Anabaena* sp. PCC 7120 and *Cyanothece* sp. ATCC 51142 are suitable. The strengths and weaknesses of these cyanobacteria are summarized in Table 2.

**Table 2:** Potential Genetic Engineering Host Strains for the Cyanobacterial Guanidine Production

Strain	N_2_-Fixation	Key Strengths	Main Weaknesses
*Synechocystis* sp. PCC 6803	No	Natural competence; thoroughly studied^66^	Moderate productivity
*Synechococcus elongatus* PCC 7942	No	Natural competence; rapid growth; high transformation efficiency^67^	Lack of guanidinase gene to protect against toxicity
*Anabaena* sp. PCC 7120	Yes	Most studied N-fixing strain	Slow growth and multicellular nature
*Cyanothece* sp. ATCC 51142	Yes	Robust and unicellular strain^68^	limited tools and difficult transformation

After implementing a conceptual design, rigorous evaluation is needed to assess system performance. The testing framework should focus on metrics such as guanidine yield, substrate flux, and secretion efficiency, employing high-throughput and quantitative assays to support iterative improvements. Beyond genetic considerations, metabolic flux analysis can identify pathway bottlenecks and guide targeted genetic engineering within the DBTL framework.

## Real-World Application of Cyanobacterial Biofertilizer

The ultimate goal is to deploy the developed cyanobacterial guanidine biofertilizer technology for agricultural utilization. There are several approaches to providing produced guanidine from cyanobacteria based on previous applications of cyanobacterial biofertilizer. First, guanidine-producing living cyanobacteria may be directly applied to the soil. As a conventional method, watering the soil with cyanobacteria or mixing cyanobacteria with vermicompost enhances crop yield.^69,70^ Pretreating of seedling roots by soaking them in cyanobacteria has also been shown to improve crop growth and yield, which appears applicable for guanidine producers through the transport of guanidine to seeds.^71^ Other methods, such as clay-based inoculation and mixing with conventional nitrogen fertilizers, may help achieving high yields for both paddy and vegetative crops by utilizing cyanobacterial guanidine.^7^ Furthermore, the application of living cyanobacteria to anaerobic rice paddy fields can mitigate methane release by methanotrophs in these systems through photosynthetic production of oxygen.^72,73^

Beyond the soil, foliar application of cyanobacterial fertilizer shows promise, as leaves are active sites of photosynthesis. Foliar nutrient application is known to be effective for nutrient absorption starting from the leaf.^74^ Additionally, it enhances health of the leaves via increasing nitrogen metabolism including nitrate reductase and glutamate synthetase,^75^ and strengthens plant immunity of maintaining the activity of the salicylic acid pathway.^76^ Foliar application of cyanobacteria increases nitrogen metabolism activity in leaves, including nitrate reductase and glutamine synthetase.^75^ Application of cyanobacteria strengthens plant immunity by maintaining the activity of the salicylic acid pathway.^77^ In hydroponic systems, addition of cyanobacteria has also improved crop yield.^78^ As outlined in this paper, further optimization of nitrogen compound production and effective application methods for cyanobacterial biofertilizer following the DBTL framework will enhance the feasibility and applications of this technology by overcoming practical challenges on scaling up and ensuring efficacy.

## Conclusions

Guanidine production from cyanobacteria represents a promising biofertilizer technology. The photosynthetic fixation of nitrogen by cyanobacteria can address the economic limitations of an energy-intensive industrial process, positioning them as powerful hosts for guanidine biofertilizer production. Strategies for achieving efficient guanidine production are proposed here for future research efforts. As an initial step, it is essential to design and identify guanidine-producing enzymes for the recombinant production of guanidine. Introducing an iron- and AKG-dependent oxygenase, an enzyme family that utilizes arginine, into nitrogen-fixing cyanobacteria shows great potential. A thorough understanding of the relevant metabolic pathways aids in identifying bottlenecks in guanidine production. Synthetic biology tools further facilitate the control of guanidine gene synthesis, and streamlining the export of guanidine from cells will enhance the efficiency of the process. Using an iterative DBTL framework to organize strategies for cyanobacterial guanidine production allows the identification of key challenges and opportunities in this emerging area.

## Supplementary Material



## Notes

S1: Concepts of Fe­(II)-and 2-oxoglutarate dependent oxygenase for guanidine production (PDF)

## References

[1] UN DESA . The Sustainable Development Goals Report 2023: Special ed. - July 2023, UN DESA: New York, USA, 2023.

[2] Alexandratos, N. ; Bruinsma, J. World agriculture towards 2030/2050: the 2012 revision; Working Paper No. 12-03; Food and Agriculture Organization of the United Nations, 2012.

[3] Zhang X., Davidson E. A., Mauzerall D. L., Searchinger T. D., Dumas P., Shen Y. (2015). Managing nitrogen for
sustainable
development. Nature.

[4] Menegat S., Ledo A., Tirado R. (2022). Greenhouse
gas emissions from global
production and use of nitrogen synthetic fertilisers in agriculture. Sci. Rep..

[5] Gao Y., Cabrera Serrenho A. (2023). Greenhouse
gas emissions from nitrogen fertilizers
could be reduced by up to one-fifth of current levels by 2050 with
combined interventions. Nature Food.

[6] Rezasoltani S., Champagne P. (2023). An integrated
approach for the phycoremediation of
Pb­(II) and the production of biofertilizer using nitrogen-fixing cyanobacteria. Journal of Hazardous Materials.

[7] Kuraganti G., Edla S., Pallaval V. B. (2020). Cyanobacteria as
biofertilizers:
current research, commercial aspects, and future challenges. Advances in Plant Microbiome and Sustainable Agriculture:
Functional Annotation and Future Challenges.

[8] Chittora D., Meena M., Barupal T., Swapnil P., Sharma K. (2020). Cyanobacteria
as a source of biofertilizers for sustainable agriculture. Biochemistry and Biophysics Reports.

[9] Sukor A., Qian Y., Davis J. G. (2023). Organic nitrogen
fertilizer selection
influences water use efficiency in drip-irrigated sweet corn. Agriculture.

[10] Liu C., Sakimoto K. K., Colón B. C., Silver P. A., Nocera D. G. (2017). Ambient
nitrogen reduction cycle using a hybrid inorganic-biological system. Proc. Natl. Acad. Sci. U. S. A..

[11] Dent, D. ; Patel, D. ; Devine, G. (2020) Microorganisms and their use in agriculture, Google Patents.

[12] Dent, D. ; Clarke, I. (2023) Plant inoculation method, Google Patents.

[13] Tamsir, A. ; Bloch, S. ; Shah, N. (2024) Gene targets for nitrogen fixation targeting for improving plant traits, Google Patents.

[14] Hessel, L. ; Shemesh, A. (2021) Process for biological ammonia production by nitrogen fixing cyanobacteria, Google Patents.

[15] Gund P. (1972). Guanidine,
trimethylenemethane, and″ Y-delocalization.″ Can acyclic
compounds have″ aromatic″ stability?. Journal of chemical education.

[16] Zhang Z., Nyborg M., Worsley M., Worsley K., Gower D. (1992). Guanidine
sulphate: slow release of mineral nitrogen during incubation in soil. Communications in soil science and plant analysis.

[17] Marsden K. A., Scowen M., Hill P. W., Jones D. L., Chadwick D. R. (2015). Plant acquisition
and metabolism of the synthetic nitrification inhibitor Dicyandiamide
and naturally-occurring guanidine from agricultural soils. Plant and Soil.

[18] Hill P. W., Marsden K. A., Jones D. L. (2013). How significant
to plant N nutrition
is the direct consumption of soil microbes by roots?. New Phytologist.

[19] Funck, D. ; Sinn, M. ; Forlani, G. ; Hartig, J. S. (2024 ) Guanidine production by plant homoarginine-6-hydroxylases, Elife 12. 10.7554/eLife.91458.3 PMC11018352 38619227

[20] Babrauskas V. (2016). Explosions
of ammonium nitrate fertilizer in storage or transportation are preventable
accidents. Journal of hazardous materials.

[21] Naeem E., Abd El-Megeed T., Emadeldin Y., Abushady A. M., Abdelrahman M. (2022). Injected anhydrous
ammonia is more effective than broadcast urea as a source of nitrogen
for drill seeded rice. Agronomy.

[22] Powlson D. S., Dawson C. J. (2022). Use of ammonium
sulphate as a sulphur fertilizer: Implications
for ammonia volatilization. Soil use and Management.

[23] Rahman N., Richards K. G., Harty M. A., Watson C. J., Carolan R., Krol D., Lanigan G. J., Forrestal P. J. (2021). Differing
effects of increasing calcium ammonium nitrate, urea and urea+ NBPT
fertiliser rates on nitrous oxide emission factors at six temperate
grassland sites in Ireland. Agriculture, Ecosystems
& Environment.

[24] Zafar N., Niazi M. B. K., Sher F., Khalid U., Jahan Z., Shah G. A., Zia M. (2021). Starch and polyvinyl alcohol encapsulated
biodegradable nanocomposites for environment friendly slow release
of urea fertilizer. Chemical Engineering Journal
Advances.

[25] Mohammadbagheri Z., Rahmati A., Hoshyarmanesh P. (2021). Synthesis
of a novel superabsorbent
with slow-release urea fertilizer using modified cellulose as a grafting
agent and flexible copolymer. Int. J. Biol.
Macromol..

[26] Chen Q., Wang Y., Zhang Z., Liu X., Li C., Ma F. (2022). Arginine increases tolerance to nitrogen deficiency in Malus hupehensis
via alterations in photosynthetic capacity and amino acids metabolism. Frontiers in plant science.

[27] Wang B., Dong T., Myrlie A., Gu L., Zhu H., Xiong W., Maness P., Zhou R., Yu J. (2019). Photosynthetic
production of the nitrogen-rich compound guanidine. Green Chem..

[28] Wang B., Xu Y., Wang X., Yuan J. S., Johnson C. H., Young J. D., Yu J. (2021). A guanidine-degrading enzyme controls genomic stability of ethylene-producing
cyanobacteria. Nat. Commun..

[29] Itzenhäuser M. A., Enkerlin A. M., Dewald J. A., Avşar B., Stauder R., Halpick H., Schaale R., Baumann L. M., Fernandez Merayo N., Maskow T., Selim K. A., Weinberg C. E., Klähn S. (2025). Deciphering guanidine assimilation
and riboswitch-based
gene regulation in cyanobacteria for synthetic biology applications. Proc. Natl. Acad. Sci. U. S. A..

[30] Kermani A. A., Macdonald C. B., Gundepudi R., Stockbridge R. B. (2018). Guanidinium
export is the primal function of SMR family transporters. Proc. Natl. Acad. Sci. U. S. A..

[31] Fukuda H., Ogawa T., Tazaki M., Nagahama K., Fujiil T., Tanase S., Morino Y. (1992). Two reactions
are simultaneously
catalyzed by a single enzyme: The arginine-dependent simultaneous
formation of two products, ethylene and succinate, from 2-oxoglutarate
by an enzyme from Pseudomonas syringae. Biochem.
Biophys. Res. Commun..

[32] Eckert C., Xu W., Xiong W., Lynch S., Ungerer J., Tao L., Gill R., Maness P.-C., Yu J. (2014). Ethylene-forming enzyme
and bioethylene production. Biotechnology for
Biofuels.

[33] Xiong W., Morgan J. A., Ungerer J., Wang B., Maness P.-C., Yu J. (2015). The plasticity of cyanobacterial
metabolism supports direct CO2 conversion
to ethylene. Nature Plants.

[34] Zhang Z., Smart T. J., Choi H., Hardy F., Lohans C. T., Abboud M. I., Richardson M. S. W., Paton R. S., McDonough M. A., Schofield C. J. (2017). Structural and stereoelectronic insights into oxygenase-catalyzed
formation of ethylene from 2-oxoglutarate. Proc.
Natl. Acad. Sci. U. S. A..

[35] Martinez S., Fellner M., Herr C. Q., Ritchie A., Hu J., Hausinger R. P. (2017). Structures and mechanisms of the non-heme Fe (II)-and
2-oxoglutarate-dependent ethylene-forming enzyme: substrate binding
creates a twist. J. Am. Chem. Soc..

[36] Ali H. S., Warwicker J., de Visser S. P. (2023). How Does the Nonheme Iron Enzyme
NapI React through l-Arginine Desaturation Rather Than Hydroxylation?
A Quantum Mechanics/Molecular Mechanics Study. ACS Catal..

[37] Durall C., Lindberg P., Yu J., Lindblad P. (2020). Increased ethylene
production by overexpressing phosphoenolpyruvate carboxylase in the
cyanobacterium SynechocystisPCC 6803. Biotechnology
for Biofuels.

[38] Scholl J., Dengler L., Bader L., Forchhammer K. (2020). Phosphoenolpyruvate
carboxylase from the cyanobacterium Synechocystis sp.PCC 6803 is under
global metabolic control by PII signaling. Molecular
microbiology.

[39] Flores E., Arévalo S., Burnat M. (2019). Cyanophycin and arginine metabolism
in cyanobacteria. Algal Research.

[40] Zhang H., Liu Y., Nie X., Liu L., Hua Q., Zhao G.-P., Yang C. (2018). The cyanobacterial
ornithine-ammonia cycle involves an arginine dihydrolase. Nat. Chem. Biol..

[41] Flores E. (2021). Studies on
the Regulation of Arginine Metabolism in Cyanobacteria Should Include
Mixotrophic Conditions. mBio.

[42] Lynch S., Eckert C., Yu J., Gill R., Maness P.-C. (2016). Overcoming
substrate limitations for improved production of ethylene in *E. coli*. Biotechnology for Biofuels.

[43] Burnat M., Picossi S., Valladares A., Herrero A., Flores E. (2019). Catabolic
pathway of arginine in Anabaena involves a novel bifunctional enzyme
that produces proline from arginine. Mol. Microbiol..

[44] Vaud S., Pearcy N., Hanževački M., Van Hagen A. M. W., Abdelrazig S., Safo L., Ehsaan M., Jonczyk M., Millat T., Craig S., Spence E., Fothergill J., Bommareddy R. R., Colin P.-Y., Twycross J., Dalby P. A., Minton N. P., Jäger C. M., Kim D.-H., Yu J., Maness P.-C., Lynch S., Eckert C. A., Conradie A., Bryan S. J. (2021). Engineering improved
ethylene production: Leveraging
systems biology and adaptive laboratory evolution. Metabolic Engineering.

[45] Schriek S., Rückert C., Staiger D., Pistorius E. K., Michel K.-P. (2007). Bioinformatic evaluation of L-arginine catabolic pathways
in 24 cyanobacteria and transcriptional analysis of genes encoding
enzymes of L-arginine catabolism in the cyanobacterium *Synechocystis
sp.*PCC 6803. BMC Genomics.

[46] Norena-Caro D. A., Zuniga C., Pete A. J., Saemundsson S. A., Donaldson M. R., Adams A. J., Dooley K. M., Zengler K., Benton M. G. (2021). Analysis of the cyanobacterial amino
acid metabolism
with a precise genome-scale metabolic reconstruction of *Anabaena
sp.*UTEX 2576. Biochemical Engineering
Journal.

[47] Greene R. F., Pace C. N. (1974). Urea and guanidine
hydrochloride denaturation of ribonuclease,
lysozyme, α-chymotrypsin, and b-lactoglobulin. J. Biol. Chem..

[48] Lenkeit F., Eckert I., Hartig J. S., Weinberg Z. (2020). Discovery
and characterization
of a fourth class of guanidine riboswitches. Nucleic acids research.

[49] Nelson J. W., Atilho R. M., Sherlock M. E., Stockbridge R. B., Breaker R. R. (2017). Metabolism of Free Guanidine in Bacteria Is Regulated
by a Widespread Riboswitch Class. Mol. Cell.

[50] Sherlock M. E., Breaker R. R. (2017). Biochemical validation
of a third guanidine riboswitch
class in bacteria. Biochemistry.

[51] Sherlock M. E., Malkowski S. N., Breaker R. R. (2017). Biochemical validation of a second
guanidine riboswitch class in bacteria. Biochemistry.

[53] Sinn M., Hauth F., Lenkeit F., Weinberg Z., Hartig J. S. (2021). Widespread
bacterial utilization of guanidine as nitrogen source. Molecular microbiology.

[54] Gordon G. C., Pfleger B. F. (2018). Regulatory tools for controlling gene expression in
cyanobacteria. Synthetic biology of cyanobacteria.

[55] Satta A., Esquirol L., Ebert B. E. (2023). Current
metabolic engineering strategies
for photosynthetic bioproduction in cyanobacteria. Microorganisms.

[56] Armshaw P., Carey D., Sheahan C., Pembroke J. T. (2015). Utilising the native
plasmid, pCA2. 4, from the cyanobacterium Synechocystis sp.strain
PCC6803 as a cloning site for enhanced product production. Biotechnology for biofuels.

[57] Ohdate K., Sakata M., Maeda K., Sakamaki Y., Nimura-Matsune K., Ohbayashi R., Hess W. R., Watanabe S. (2024). Discovery
of novel
replication proteins for large plasmids in cyanobacteria and their
potential applications in genetic engineering. Frontiers in Microbiology.

[58] Koblenz B., Lechtreck K.-F. (2005). The NIT1 promoter allows inducible and reversible silencing
of centrin in Chlamydomonas reinhardtii*Chlamydomonas reinhardtii*. Eukaryotic cell.

[59] Bolay P., Schlüter S., Grimm S., Riediger M., Hess W. R., Klähn S. (2022). The transcriptional
regulator RbcR controls ribulose-1,
5-bisphosphate carboxylase/oxygenase (RuBisCO) genes in the cyanobacterium *Synechocystis sp.* PCC 6803. New Phytologist.

[60] Moore V., Vermaas W. (2024). Functional consequences
of modification of the photosystem
I/photosystem II ratio in the cyanobacterium *Synechocystis
sp.* PCC 6803. Journal of bacteriology.

[61] Guerrero F., Carbonell V., Cossu M., Correddu D., Jones P. R. (2012). Ethylene
synthesis and regulated expression of recombinant protein in *Synechocystis sp.* PCC 6803. PloS one.

[62] Lan E. I., Liao J. C. (2011). Metabolic engineering
of cyanobacteria for 1-butanol
production from carbon dioxide. Metabolic engineering.

[63] Liu D., Pakrasi H. B. (2018). Exploring native
genetic elements as plug-in tools
for synthetic biology in the cyanobacterium *Synechocystis
sp.*PCC 6803. Microbial Cell Factories.

[64] Salis H. M., Mirsky E. A., Voigt C. A. (2009). Automated
design of synthetic ribosome
binding sites to control protein expression. Nature biotechnology.

[65] Seo S. W., Yang J.-S., Kim I., Yang J., Min B. E., Kim S., Jung G. Y. (2013). Predictive design of mRNA translation initiation region
to control prokaryotic translation efficiency. Metabolic engineering.

[66] Barten R., Lill H. (1995). DNA-uptake in the naturally competent
cyanobacterium,*Synechocystis
sp.*PCC 6803. FEMS Microbiology Letters.

[67] Taton A., Erikson C., Yang Y., Rubin B. E., Rifkin S. A., Golden J. W., Golden S. S. (2020). The circadian
clock and darkness
control natural competence in cyanobacteria. Nat. Commun..

[68] Liu D., Liberton M., Yu J., Pakrasi H. B., Bhattacharyya-Pakrasi M. (2018). Engineering
nitrogen fixation activity in an oxygenic phototroph. MBio.

[69] Alharbi K., Hafez E. M., Omara A. E.-D., Osman H. S. (2023). Mitigating osmotic
stress and enhancing developmental productivity processes in cotton
through integrative use of vermicompost and cyanobacteria. Plants.

[70] Massey M. S., Davis J. G. (2023). Beyond soil inoculation:
Cyanobacteria as a fertilizer
replacement. Nitrogen.

[71] Chittapun, S. ; Lomphengthian, K. ; Amnuaysin, N. ; Piyapittayanun, C. Improvement of rice growth and yield by seedling pretreatment to induce the artificial coexistence of nitrogen-fixing cyanobacteria and root seedling, ScienceAsia 2024, 50. 10.2306/scienceasia1513-1874.2024.020

[72] Pandey P., Pandey D., Gupta A., Gupta R., Tiwari S., Singh S. P. (2025). Cyanobacterial green
chemistry: a blue-green approach
for a sustainable environment, energy, and chemical production. RSC Sustainability.

[73] Prasanna R., Kumar V., Kumar S., Kumar Yadav A., Tripathi U., Kumar Singh A., Jain M. C., Gupta P., Singh P. K., Sethunathan N. (2002). Methane production in rice soil is
inhibited by cyanobacteria. Microbiological
Research.

[74] Godlewska K., Michalak I., Pacyga P., Baśladyńska S., Chojnacka K. (2019). Potential applications of cyanobacteria: Spirulina
platensis filtrates and homogenates in agriculture. World J. Microbiol. Biotechnol..

[75] Bhardwaj A., Prasanna R., Bavana N., Kokila V., Nivedha R. M., Gaur Rudra S., Singh A. K., Shivay Y. S. (2025). Foliar
application
of cyanobacterial formulations stimulates plant growth and fruit quality
in tomato under protected cultivation. New Zealand
Journal of Crop and Horticultural Science.

[76] Abdelaziz A. M., Attia M. S., Salem M. S., Refaay D. A., Alhoqail W. A., Senousy H. H. (2022). Cyanobacteria-mediated
immune responses in pepper plants
against fusarium wilt. Plants.

[77] Toribio A. J., Jurado M. M., Suárez-Estrella F., López-González J. A., Martínez-Gallardo M. R., López M. J. (2021). Application
of sonicated extracts of cyanobacteria and microalgae for the mitigation
of bacterial canker in tomato seedlings. Journal
of Applied Phycology.

[78] Kholssi R., Marks E. A., Miñón J., Maté A. P., Sacristán G., Montero O., Debdoubi A., Rad C. (2021). A consortium
of cyanobacteria and plant growth promoting rhizobacteria for wheat
growth improvement in a hydroponic system. South
African journal of botany.

