# Supplementary material for: Cyanobacterial Biofertilizer Production by Guanidine-Producing Enzymes
Source: ACS Synth Biol. 2026 Feb 10;15(2):359–67. doi: 10.1021/acssynbio.5c00801 (PMC12930504; doi:10.1021/acssynbio.5c00801)
Supplement: Supplementary file 1 [file sb5c00801_si_003.pdf]

Cyanobacterial Biofertilizer Production by Guanidine-Producing Enzymes

Hakyung Lee[1,2], Jacob Sebesta[1], Eric Schaedig[1], Chao Wu[1], Himadri B. Pakrasi[2], Jianping Yu[1,*]

[1]Bioscience Center, National Laboratory of the Rockies, Golden, CO 80401, United States

[2]Washington University, Saint Louis, MO 63130, United States

[*]Correspondence: Jianping.Yu@nlr.gov (Jianping Yu).

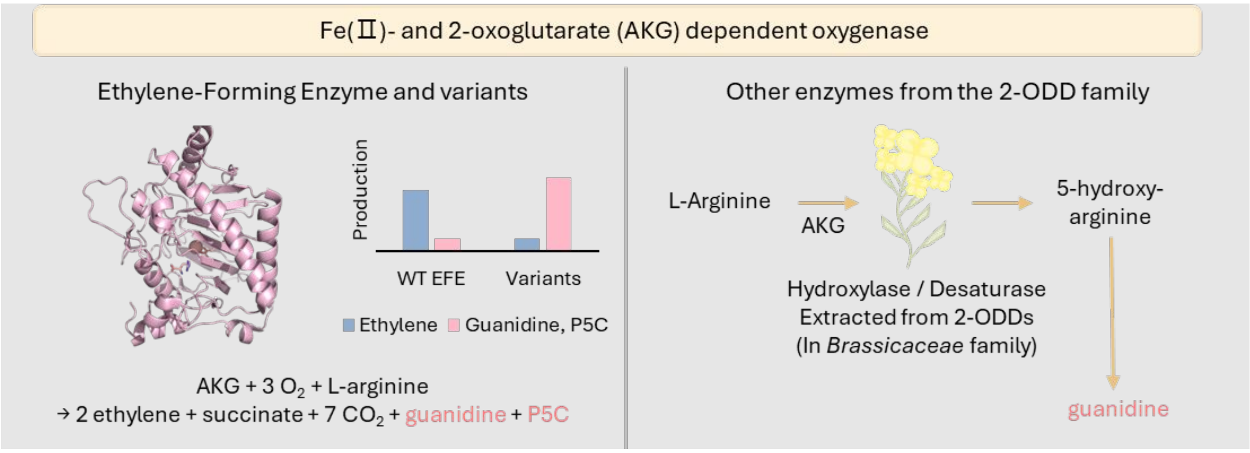

Figure S1. Concepts for designing and determining potential guanidine-producing enzymes based on the characteristics of Fe (Ⅱ)- and 2-oxoglutarate (AKG) dependent oxygenase family including ethylene-forming enzyme monomer in *Pseudomonas syringae.*
